# Low Level of Autophagy-Related Gene 10 (*ATG10*) Expression in the 6-Hydroxydopamine Rat Model of Parkinson’s Disease

**DOI:** 10.22034/ibj.22.1.15

**Published:** 2018-01

**Authors:** Marzieh Shams Nooraei, Ali Noori-Zadeh, Shahram Darabi, Farzad Rajaei, Zohreh Golmohammadi, Hojjat Allah Abbaszadeh

**Affiliations:** 1 Cellular and Molecular Research Center, Qazvin University of Medical Sciences, Qazvin, Iran; 2 Department of Clinical Biochemistry, Faculty of Medicine, Ilam University of Medical Sciences, Ilam, Iran; 3 Hearing Disorder Research center, Shahid Beheshti University of Medical Science, Tehran, Iran

**Keywords:** Parkinson’s disease, Autophagy, 6-hydroxydopamine (6-OHDA), ATG16L, ATG10

## Abstract

**Background:**

Autophagy is a mechanism disassembling the damaged organelles from the cell. This study attempted to examine the expression of several autophagy-related genes in Parkinson’s disease (PD) rat model.

**Methods:**

The male Wistar rats were divided into three groups as control, sham, and lesion. In the latter group, the PD rat model was induced by the injection of 6-hydroxydopamine in the striatum. The behavioral test was conducted one (baseline) and four weeks after the surgery through apomorphine hydrochloride. Then the RT-PCR technique was employed to evaluate the expressions of *p62/SQSTM*, autophagy-related genes *(ATG)5*, *ATG12*, *ATG16L1*, *ATG10*, as well as *GAPDH* and *LC3*.

**Results:**

By injecting apomorphine, the striatal lesion group showed a significant contralateral rotation at fourth week as compared to the baseline. The examination of *p62*, *ATG5*, *ATG12*, *ATG16L1*, and *LC3* expressions using RT-PCR revealed that *p62*, *ATG5*, *ATG12*, *LC3*, and *ATG16L1* were expressed in the substantia nigra of PD rat model, while *ATG10* was not expressed.

**Conclusion:**

ATG10 expression is necessary for the initiation of autophagy. Thus, these results show that autophagy deregulation occurs in the initiation stages of the process in the rat model of PD.

## INTRODUCTION

Parkinson’s disease (PD) affects the central nervous system in adults at later ages[1]. The loss of dopaminergic neurons in the substantia nigra is responsible for PD motor symptoms[2]. PD is characterized based on the aggregation of alpha-synuclein protein, as a constituent compound of Lewy bodies, at the cellular and molecular levels[3,4]. The genetic causes of PD have been identified for about 10% of the cases[5]. Evidence suggests that mitochondrial dysfunction contributes to protein aggregation and autophagic stress in the pathogenesis of neurodegenerative diseases[6].

Autophagy is a physiological process playing an important role in cell homeostasis through the digestion of damaged proteins and organelles[7]. Numerous studies suggest that impaired autophagy leads to aging and neurodegenerative diseases such as PD[8-14], Alzheimer’s[15], and Huntington’s disease[16].

In this regard, the executers of autophagy, including *p62/SQSTM*,*LC3* and autophagy-related genes (*ATG*) such as *ATG5*, *ATG12*, *ATG16L1*, and *ATG10* are associated with PD development[17-21]. ATG10 contributes to autophagosome formation by interacting with ATG7 to receive ATG1, which is an ubiquitin-like molecule. Moreover, it contributes to the formation of autophagosome in the reaction of ATG5-ATG12 conjugation[22]. More details about ATG10 roles in autophagy can be found in recent studies[23,24].

Although, it is crucial to identify the *ATG* s and their links with PD; however, little is known about gene expression alterations of these genes in the course of the disease development. Using unilateral 6-hydroxydopamine (6-OHDA)-induced Wistar rat model of PD, we investigated this phenomenon.

## MATERIALS AND METHODS

### Experimental procedure

A total number of 30 male Wistar rats, weighing 200-250 g, was supplied by Razi Vaccine and Serum Research Institute (RVSRI) based in Karaj, Iran. The animals were caged in the groups of 4 to 6, and maintained in an environment with identical lighting conditions at 23-25 °C. There was no restriction to food/water access for all animals. Before surgery, the rats were anesthetized with a combination of ketamine (100 mg/kg, i.p.) and xylasine (5 mg/kg, i.p.). All efforts were made to minimize animal suffering and the procedures carried out with institutional ethical approval and according to the Animal Care and Ethics Committee of Qazvin University of medical science (Qazvin, Iran), which is in agreement with the guidelines of the national institutes of health for the use of live animals.

The rats without any sign of biased rotational behavior (net rotations less than 30 route per hour) following i.p. injection of apomorphine hydrochloride (0.5 mg/kg) were selected for the present study[25]. Then they were randomly divided into three groups, 10 rats in each group: control (no injection), sham (0.9% normal saline with 0.2 mg/ml ascorbic acid into the left striatum), and lesion (unilateral stereotaxic injection of 5 µl neurotoxin solution containing 2.5 μg/kg of 6-OHDA [Sigma, Germany] dissolved in 0.9% normal saline with 0.2 mg/ml of ascorbic acid into the left striatum to induce striatal lesioned rat model of PD). The injection rate was one microliter per minute in all groups with injections. Left striatum injections were performed through a 5-µl Hamilton syringe on anesthetized rats using stereotaxic apparatus (Stoelting, USA) with coordinates of 3 mm lateral to the left, +9.2 mm interaural (anteroposterior proportion to the distance between the ears), and 4.5 mm dorsoventral from the dura mater surface. The incisor bar was placed 3.3 mm below the horizontal plane. The stereotaxic apparatus was set up according to the Paxinos and Watson’s atlas[26]. Five minutes after the injections, the syringe was slowly withdrawn to prevent back filling along the injection tract and also diffusion of the toxin away from the injection site, and then retracted. After surgery, the head skin was sutured (stitched) and it was disinfected to prevent any infections. The control group received no injection.

### Behavioral study

The behavioral tests were conducted in two phases: before and four weeks after developing the PD rat model. For assessing behavioral test, 0.5 mg/kg of apomorphine hydrochloride (Sigma, St. Louis, MO) was injected intraperitoneally. Dopamine agonists such as apomorphine are effective in measuring rotational asymmetry in unilateral striatal-lesioned animals. This measurement was analyzed by placing rats in a white hemispheric plastic rotation bowl (35 cm wide at top and 33 cm diameter). At the next stage, the number of full 360-degree rotations toward the lesion’s opposite side was counted manually for 60 minutes in a quiet isolated room. The number of contra-lateral rotations was counted as positive scores for apomorphine. Net number of rotations was defined as the positive scores (contra-lateral rotations) minus the negative scores (ipsi-lateral rotations)[27].

### RT-PCR

Briefly, after behavioral tests, the rats were anaesthetized with ketamine (100 mg/kg, i.p.) and xylasine (5 mg/kg, i.p.) and 500 µm sections were cut with a slicer. The substantia nigra pars compacta were micropounched from the slices and the neurons dissociated mechanically with a scalpel, and total RNA was extracted using a total RNA extraction kit supplied by Roche Biochemicals (Germany). The unwanted DNA was removed by DNase I treatment kit (Fermentas, USA). Then, the RNA was converted into cDNA using a cDNA synthesis kit (Fermentas, USA) by reverse transcriptase enzyme. At the next stage, the cDNA was amplified using PCR and finally examined by gel electrophoresis method. Table 1 displays the examined genes and their corresponding primers for each of them.

**Table 1:** Genes and their corresponding primers used for the RT-PCR reactions

Gene	Accession number	Primer sequence (5’◊3’)	Fragment size (bp)
*p62*	113894	TCCTACAGACCAAGAATTATGAC	232
		TTCTCATGCACTTTCCTACTG	
*ATG10*	688555	CCTGTTTGCTTGGGATAGTGG	174
		ACTTCCCCATCAATCTCCAC	
*ATG5*	365601	CCTGAAGACGGAGAGAAGAAGAG	215
		CGGGAAGCAAGGGTGTCAT	
*ATG12*	361321	CATTCTTACCTGGCGTTGAG	168
		CACTTCAAACCCTGTAATCC	
*ATG16L1*	363278	CACATCTTTACCCAGCATCAC	Fragment size bp
		CAGGACAGAGGGTGCTTTC	
*LC3*	25291	TGTTAGGCTTGCTCTTTTGG	232
		GCAGAGGAAATGACCACAGAT	

## RESULTS

### Behaverial tests

**Fig. 1:**
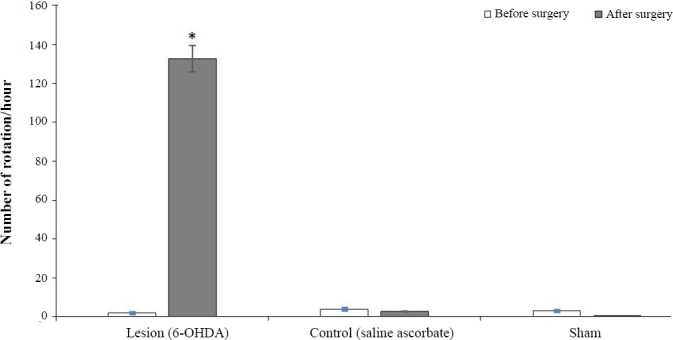
Behavioral test results in the three experimental groups. The Figure displays the total number of rotations during one hour after apomorphine injection in a week, before and four weeks after surgery. There were no significant differences between the control and experimental groups regarding the number of rotations induced during four weeks after surgery and a week before it. In the lesion group (unilateral stereotaxic injection of 5 µl neurotoxin solution containing 2.5 μg/kg of 6-hydroxydopamine i.e. 6-OHDA, dissolved in 0.9% normal saline with 0.2 mg/ml of ascorbic acidinto the left striatum), the number of the contralateral rotations indicated a significant difference between four weeks after and one week before surgery (^*^ p < 0001).

No significant differences were observed between the control and sham groups regarding the number of rotations induced by i.p. injection of apomorphine four weeks after the surgery in comparison with one week before it (Fig. 1). Following apomorphine injection, the rats of the lesion group showed a significant number of contralateral rotations (*p* < 0001) after 4 weeks as compared to one week before the surgery. Moreover, the mean number of rotations was 132.75. This observation indicated a significant difference from the other two groups, reflecting the successful development of PD model in the lesion group.

### Gene expression

**Fig. 2:**
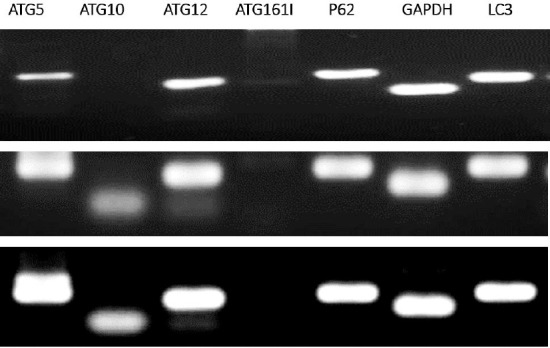
The RT-PCR results in three experimental groups. The rows represent the mRNA expression results for *p62*, *ATG5*, *ATG10*, *ATG12*, *ATG16L1*, *LC3*, and *GAPDH* in the rat model of Parkinson’s disease (row 1), control (row 2), and sham (row 3) groups. Genes of *p62*, *ATG5*, *ATG12*, *LC3*, *ATG16L1(very faint band seen)*, and *GAPDH* were expressed in the Parkinson’s disease rat model, whereas *ATG10* was not expressed. Besides, unlike *ATG10*, *ATG16L1* was not expressed in the control and sham groups.

Using RT-PCR method, the expressions of *p62*, *ATG5*, *ATG10*, *ATG12*, *ATG16L1*, LC3, and *GAPDH* in the rat model of PD as well as in the control and sham groups were examined. It was observed that *p62*, *ATG5*, *ATG12*, *LC3*, and *GAPDH* were expressed in three experimental groups. In one hand, *ATG10* and *ATG16L1* were not expressed in the PD rat model, and on the other hand, *ATG10* was only expressed in the control and sham groups. *GAPDH* gene was used as an internal control (Fig. 2).

## DISCUSSION

The rat PD model was developed by injecting 6-OHDA toxin intrastriatally[28]. Following the injection of 6-OHDA into the striatum, there was a long-term, persistent and a distinct decline in the number of neurons in the substantia nigra on the lesion side.

Neurotoxin 6-OHDA damages the dopaminergic nigrostriatal pathway by inducing the production of hydrogen peroxide and the derivative highly active hydroxyl free radicals, probably in the presence of iron[29,30]. In this study, the PD model was developed by injecting 12.5 µg 6-OHDA toxin in 5 µl saline ascorbate solution into the left striatum. Due to the toxin injection, the dopaminergic neurons were partially destroyed in nigra pars compacta[28]. This model largely resembles the onset of PD in humans. Such unidirectional changes lead to a motor asymmetry measurable quantitatively using the dopaminergic agonists such as apomorphine, which directly affects the dopamine D1- and D2-like receptors[31].

**Fig. 3:**
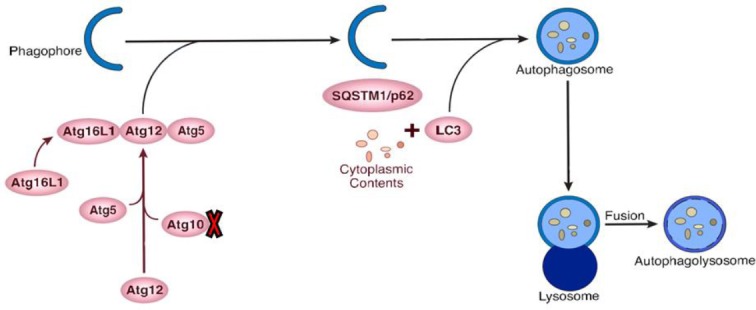
Schematic representation of autophagy-related gene proteins involved in the initiation and elongation of the autophagosome formation. The expression rates of *ATG10* was decreased in the rat model of Parkinson’s disease, as shown by a red mark.

Apomorphine induces the rotation toward the right side of animals by destructing the left side of striatum[32]. The lesion group of PD rat model showed a significant number of rotations toward the right side as a result of apomorphine injection intraperitoneally after four weeks, compared to one week before the surgery. This result reflected that the rats developed a PD-like disease, destructing their nigrostriatal system. It is well accepted that the dopaminergic neurotoxicant 6-OHDA induces oxidative damage in cell culture and animal models of PD. Upon oxidative damage to the cell components such as proteins, autophagy is induced to degrade unfolded or misfolded proteins. These observations imply that autophagy plays an important role in the pathogenesis of the disaese. After establishing the rat model of PD, we explored the *ATG* gene expression levels in three developed groups using RT-PCR. Interestingly, the results showed that *p62*, *ATG5*, *ATG12*, *LC3*, and *GAPDH* genes were expressed in the rat model of PD, whereas *ATG10* and *ATG16L1* genes were not expressed. Also, *ATG10*, but not *ATG16L1*, was expressed in the control and sham groups (Fig. 3).

One of the key results of our investigation was *ATG16L1* expression in the rat model of PD. It is known that autophagy factor ATG12-ATG5 conjugate exhibits E3 ligase-like activity, which facilitates the lipidation of members of the LC3 family. This conjugate interacts with ATG16L1. The latter factor recruits the conjugate to autophagosomal membranes to expand the nascent autophagosomes. *ATG16L1* was not expressed in both sham and control groups; however, its expression was inducble and actually increased in the rat model of PD in comparison with the two other groups. As mentioned above, due to the low/absence of *ATG10* expression, autophagy performance quality may be hampered; nevertheless, *ATG16L1* is overexpressed and exists at the proper level in the dopaminergic cells in the rat model of PD. This information points out that autophagy dysregulation in the rat model of PD may be a consequence of low or no expression of ATG10 and also low or absence of ATG12-ATG5 conjugate activity for autophagosome formation (Fig. 3).

p62/SQSMT1/A170 is an intracellular protein contributing to the cell survival and death by stress and regulating various signal transduction pathways. p62 binds the ubiquitinated proteins to LC3 for degradation through autophagy[33]. When autophagy is blocked up, the p62 accumulation escalates, serving as a marker of autophagy[19,21]. p62 is an ubiquitin-binding protein that interacts with LC3, playing an important role in transporting the autophagic load to phagophore[21]. p62 is required for ubiquitinated proteins that have a vital function in autphagic clearing. Moreover, it releases Beclin1 by rupturing the Beclin1-Bcl2 bond, thereby positively regulating the induction of autophagy[21].

LC3 (ATG8) splits adjasent to the Gly 120 shortly after synthesis in the cytoplasm, leading to the production of LC3 I. Then a subset of LC3 I converts into LC3 II, which concentrates on the autophagosomal membranes ofautophagy. The LC3 conversion can be used to control autophagic activity. The formation of LC3II depends on ATG5 binding to ATG12[34,35]. In this investigation, the two genes, *ATG5* and *ATG12*, were expressed. Hence, it is likely that LC3 converts into LC3 II. LC3 is involved in the elongation stage of autophagy, and it was expressed in all three groups of rats.

During elongation, conjugated ATG8- phosphatidylethanolamine (PE) sits on the phagophore membrane contributes to load transportation into the autophagosome. ATG12 and ATG5 are conjugated through an ubiquitination-like mechanism. At first, glycine is activated at the carboxyl terminal chain of ATG12 by ATG7 (E1-like enzyme) through ATP-dependent high-energy bonds. ATG12 is transported to ATG10 (E2-like enzyme), which then bonds to lysine 149 of ATG5. The transport of LC3 from ATG8 to PE is stimulated by a complex of ATG5-ATG12-ATG16L1, specifying where LC3-PE is produced[36]. The ATG5-ATG12 conjugation interacts with ATG16L1 to form ATG5-ATG12-ATG16L1. Thus, the lipidation system, ATG5-ATG12-ATG16L1, is essential for the formation of autophagosome.

As mentioned earlier, ATG10 contributes to the formation of autophagosome. It interacts with ATG7 to receive ATG1, which is an ubiquitin-like molecule. Moreover, it is involved in the formation of autophagosome in the reaction of ATG5-ATG12 conjugation[22]. Jo *et al*.[37] argued that the expression of ATG10 has been observed to be associated with metastasis of lymph node and lymphatic vessels in rectal cancer.

ATG16L1 takes an essential part in autophagy process. It interacts with ATG5-ATG12 or PE-conjugated with LC3, converting the active form of LC3 to LC3 II, thereby to control the spread of autophagosome membrane[38]. ATG16/ ATG16L1 is essential for autophagy because it regulates the localization of ATG5-ATG12 in yeast. Moreover, ATG16L1 interacts with FIP200 to correctly target ATG16L1 complex where autophagosome forms. In the cells evacuated from FIP200, the staining of ATG16L1 indicates a scattered cytoplasmic pattern[39]. A study proved that the majority of rats with deficient ATG16L1 died one day after birth. This result indicates that ATG16L1 is vital for survival during neonatal starvation, and LC3-PE bond is rarely seen in such rats[40]. Complex ATG5-ATG12 was also rarely observed in the fibroblasts of rats with deficient ATG16L1. Moreover, the autophagosome cannot form, thereby leading to aggregation of long-lived proteins and p62[41]. *ATG10* down-regulation in the rat model of PD was the main result of this investigation. Whether this gene is down-regulated in other models, or how 6-OHDA can down-regulate this gene or how disrupted autophagy could lead to PD development is the matter of debate and merits further investigations.

In PD, ubiquitinated proteins aggregate. Meanwhile, the expression of *p62* seems to be involved in protein aggregation in PD as well as their clearing through autophagy. The expression of *LC3* demonstrated that p62 in PD can interact with LC3, which has a key role in transporting the autophagic load into phagophore. The findings suggested no expressions of *ATG10*; hence, ATG5 and ATG12 cannot bind. Furthermore, it was revealed that ATG16L1 is crucial for autophagy in regulating the localization of ATG5-ATG12. As a consequence of *ATG10* down-regulation, LC3 cannot bind to PE. In fact, autophagy is unregulated and incomplete in elongation.
